# Growth-Promoting Treatment Screening for Corticospinal Neurons in Mouse and Man

**DOI:** 10.1007/s10571-020-00820-7

**Published:** 2020-03-14

**Authors:** Nicholas Hanuscheck, Andrea Schnatz, Carine Thalman, Steffen Lerch, Yvonne Gärtner, Micaela Domingues, Lynn Bitar, Robert Nitsch, Frauke Zipp, Christina F. Vogelaar

**Affiliations:** 1 Department of Neurology, Focus Program Translational Neuroscience (FTN) and Immunotherapy (FZI), Rhine Main Neuroscience Network (rmn2), University Medical Center of the Johannes Gutenberg University Mainz, 55131 Mainz, Germany; 2 Institute for Developmental Biology and Neurobiology, Molecular Cell Biology, Johannes Gutenberg University Mainz, 55099 Mainz, Germany; 3 University Medical Center, Institute for Translational Neuroscience, Westfälische Wilhelms-University Münster, Albert-Schweitzer-Campus, 48149 Münster, Germany

**Keywords:** Corticospinal tract, Interleukin-4, Growth-promoting treatment, Regeneration, Spinal cord injury

## Abstract

Neurons of the central nervous system (CNS) that project long axons into the spinal cord have a poor axon regenerative capacity compared to neurons of the peripheral nervous system. The corticospinal tract (CST) is particularly notorious for its poor regeneration. Because of this, traumatic spinal cord injury (SCI) is a devastating condition that remains as yet uncured. Based on our recent observations that direct neuronal interleukin-4 (IL-4) signaling leads to repair of axonal swellings and beneficial effects in neuroinflammation, we hypothesized that IL-4 acts directly on the CST. Here, we developed a tissue culture model for CST regeneration and found that IL-4 promoted new growth cone formation after axon transection. Most importantly, IL-4 directly increased the regenerative capacity of both murine and human CST axons, which corroborates its regenerative effects in CNS damage. Overall, these findings serve as proof-of-concept that our CST regeneration model is suitable for fast screening of new treatments for SCI.

**Electronic supplementary material:**

The online version of this article (10.1007/s10571-020-00820-7) contains supplementary material, which is available to authorized users.

## Introduction

Axon tracts in the spinal cord regenerate poorly, with the corticospinal tract (CST) being the least effective regenerator (Schiwy et al. 2009). This is thought to be due to neuron-intrinsic as well as extrinsic factors in the lesioned spinal cord (Tedeschi and Bradke 2016; Vogelaar 2016). Not only growth-inhibitory molecules in and around the lesion are a barrier to axon regeneration, but also intrinsic regenerative responses, like the fast formation of a new growth cone and the activation of a regeneration program, are principally lacking in CNS neurons (Bradke et al. 2012; Chew et al. 2012; Mason et al. 2003; Verma et al. 2005; Vogelaar 2016). Especially these intrinsic regeneration mechanisms are interesting targets for regeneration-promoting treatments, since many treatments exist that reduce the inhibitory barrier, but the lack of intrinsic axon outgrowth is still a great limiting factor (Bradke et al. 2012; Bunge 2008). Testing of putative treatments is mostly performed in animal SCI models, is time-consuming and expensive and involves the use of large numbers of animals (Abu-Rub et al. 2010; Robins and Fehlings 2008; Vogelaar and Estrada 2016). An in vitro model for CST regeneration would greatly accelerate the screening for treatments that increase the intrinsic regenerative capacity of CST axons.

Here, we used motor cortex layer V (CxV) explant cultures to grow CST axons in vitro and in parallel performed dorsal root ganglion (DRG) cultures to directly compare PNS and CNS axons with regard to their morphology and growth (Vogelaar et al. 2009). We developed a regeneration assay to assess the regeneration-promoting efficacy of putative treatments.

In the past few decades, the concept of neuro-immune interactions has been gaining ground (Ellwardt et al. 2016; Kipnis and Filiano 2018). Especially a beneficial role of T cells in central nervous system (CNS) injury is now well-established (Schwartz and Kipnis 2001). Recently, we discovered a neuron-specific fast direct interleukin-4 receptor (IL-4R) signaling pathway leading to neuroprotection (Vogelaar et al. 2018; Walsh et al. 2015). These observations led to the hypothesis that IL-4 may act directly on the CST. Using insulin-like growth factor-1 (IGF-1) as a proof-of-principle and IL-4 as test substance, we validated the CST regeneration assay and found that IL-4 significantly increased the formation of new growth cones after axon transection. Combined with IL-4 effects on outgrowth in human neuronal cultures, these models provide evidence for the direct regenerative effects of IL-4 and are suitable for the screening of putative growth-promoting treatments for traumatic CNS injury.

## Materials and Methods

### Animals

Pregnant C57BL/6 mice were purchased from Janvier and C57BL/6 Thy1-YFP-H (Carter et al. 2008) and Bl6-GFP mice (C57BL/6-Tg(CAG-EGFP)1Osb/J; (Feng et al. 2000; Ikawa et al. 1998)) were obtained from The Jackson Laboratory. All animals were kept under pathogen-free conditions in individually ventilated cages with ad lib access to food and water at all times. Animals were killed in accordance with §4 of the German Animal Welfare Act.

### DRG Explant Culture

DRGs were isolated using previously described protocols (Vogelaar et al. 2009). Briefly, the spinal column of adult mice was opened via laminectomy and DRGs were pulled out of the intervertebral foramina. After removing nerve stumps and connective tissue, two DRG explants per well were plated in 4-well-Nunclon plates (Thermo scientific) coated with poly-D-lysine (PDL; 0.5 mg/ml; Sigma) and laminin (1 µg/ml; Life Technologies). Growth medium consisted of neurobasal medium containing 2% horse serum (Vector Laboratories), 1% Glutamax, 2% B-27 and 1% penicillin/streptomycin (all Life Technologies), supplemented with 10 ng/ml nerve growth factor (NGF, Sigma). Explants were cultivated for 4–6 days in an incubator at 37 °C with 5% CO_2_.

### CxV Explant Culture

**Fig. 1:**
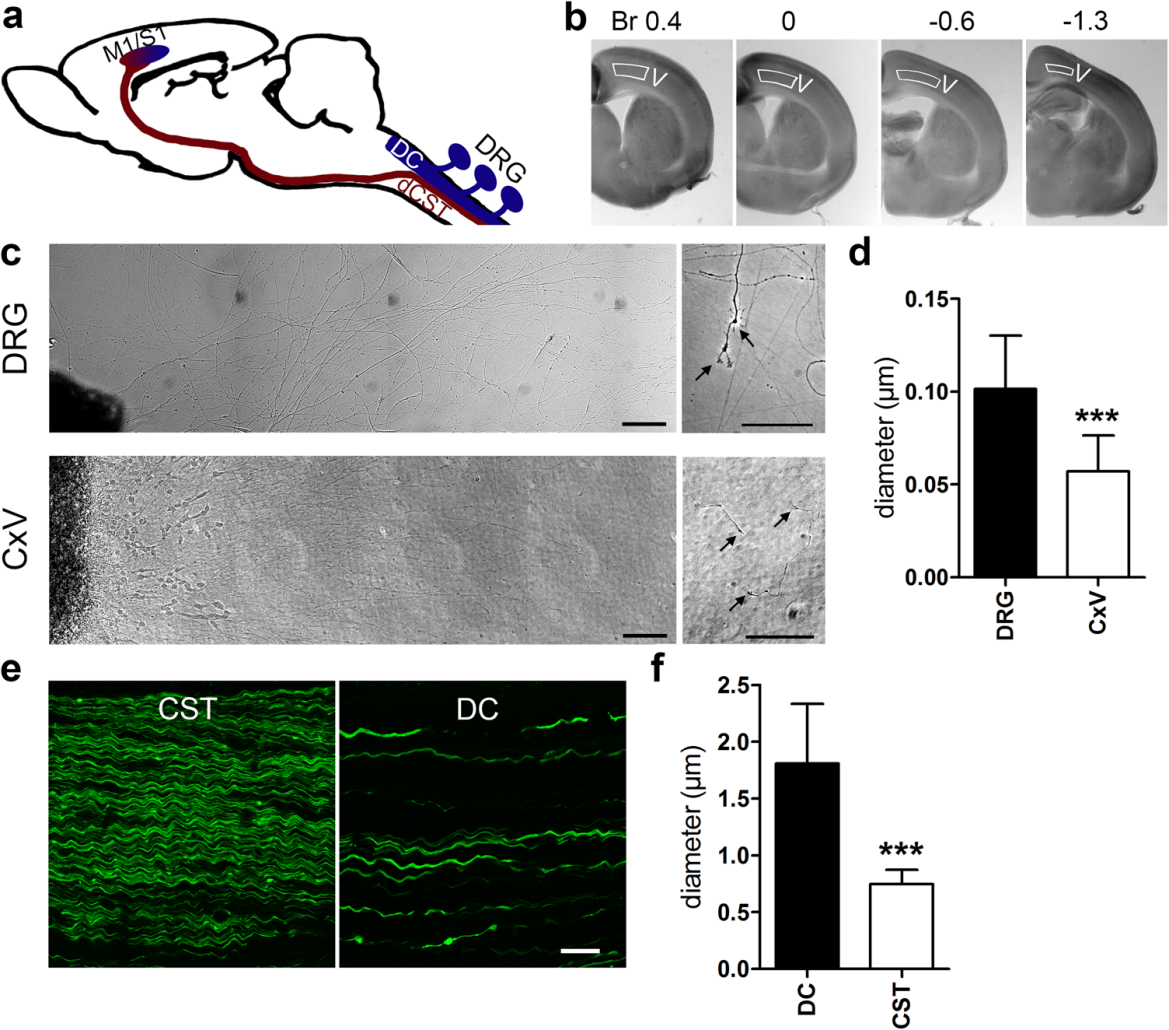
Preparation and characterization of CxV explants. **a** Schematic drawing of the mouse CNS, with dorsal root ganglia (DRGs) depicted on the side of the spinal cord. Pyramidal motor neurons project their axons from primary motor cortex (M1) through the pyramidal tract into the dorsal corticospinal tract (dCST). Fibers from the DRGs ascend through the dorsal columns (DC, DRG) to the hindbrain. Sensory information is directed through several relays to the primary sensory cortex (S1). In the mouse, M1 and S1 overlap and are therefore mostly referred to as sensorimotor cortex. **b** Coronal 250 µm vibratome slices of a P1 mouse brain at indicated Bregma positions 0.4 to − 1.3 used for the dissection of motor cortex layer V (CxV) explants (delineated regions). Explants were divided in two or three pieces and subsequently cultivated. **c** Brightfield microscopic images of DRG (upper panel) and CxV (lower panel) explants show robust neurite outgrowth and stable growth cone formation (right panels) after 3 days in vitro. Axons and growth cones of the cortex explants were smaller in diameter compared to those of the DRGs. **d** Quantification of axon diameter of DRG and CxV axons in culture (*n* = 10). **e** Parasagittal spinal cord section of a YFP-H mouse with YFP-labeled axons of the DC and the CST. **f** Quantification of axon diameter of DC and CST axons in vivo (*n* = 10). Statistics: *t*-test, ***p < 0.001. Scale bars: (**c**) 100 µm, (**e**) 25 µm

Previously described protocols for short-term cultures of cortex or hippocampus (Liu et al. 2005; Niquille et al. 2009) were modified to establish long-lasting cultures of motor cortex layer V explants. Brains from neonatal mice (P0–P5) were transferred to tissue-embedding molds filled with ~ 37 °C warm low melting point agarose (3%, Invitrogen) in sterile dissecting medium (1 × MEM (Gibco), 10 mM Tris (Roth), 30 mM dextrose (Sigma)). Brains were cut in 250 μm-thick coronal slices corresponding to Br − 0.5 to − 1.3 (sensorimotor cortex) using a vibratome (HM650V, Thermo Scientific). Layer V of the motor cortex was dissected (Fig. 1a–c) and divided in 2–3 explants that were plated in 4-well-Nunclon plates (Thermo scientific) coated with PDL-laminin and cultivated in growth medium (see above). Explants were kept in a low level of culturing medium and shear forces were avoided to prevent explants from detaching. Explants were grown for up to 2 weeks ensuring growth of long axons (Suppl. Fig. S1).

### CxV Growth Assay

Cortex explants that grew quantifiable axons within 24 h were treated for another 24 h with 50 ng/ml IGF-1, 50 ng/ml IL-4, or phosphate buffered saline (PBS) as control. Images were made daily using the Olympus IX51 and CellSense Dimension software (Olympus, XC30). Axon length was assessed using Photoshop (Adobe) by drawing straight lines from the 40 longest axons to the explants (90° angle). The growth at 48 h (1 day after treatment) was corrected for the initial growth at 24 h.

### In vitro Transection

After 5–7 days in vitro (div), selected CxV explants were subjected to in vitro transection using a sterile 0.125 mm-thick Tungsten needle (Fine Science Tools). The axons were transected at ~ 1 mm distance to the explants (see Suppl. Fig. S2). Explants were subjected to treatment with 50 ng/ml IGF-1 (Peprotech), 50 ng/ml recombinant mouse (r-m) IL-4 (Peprotech), or equal volumes of PBS. For scoring degeneration, retraction and regeneration, images were made with the Olympus IX51 microscope and CellSense Dimension software (Olympus, XC30) at 0, 90 and 180 min post-transection. For immunocytochemistry, the cultures (on coverslips) were fixed 120 min post-transection.

### Human Neural Stem Differentiation and Neuron Growth Assay

H9-derived human neural stem cells (H9 hNSCs, Gibco) were cultured according to the manufacturer’s instructions. Briefly, H9 hNSCs were expanded and subsequently differentiated in neurobasal medium, 2% B27, 1% Glutamax, and 1% penicillin/streptomycin, supplemented with 10 ng/ml BDNF (Peprotech), and 2 ng/ml recombinant human GDNF (Peprotech). After 7 days, 500 µM of db-cAMP (N6,2′-*O*-dibutyryladenosine 3′,5′-cyclic monophosphate, Sigma) was added. Cells were fixed with 4% paraformaldehyde (PFA, Sigma) for immunocytochemistry at 3 weeks post-differentiation. For assessing neurite outgrowth, hNSCs were differentiated for 6 days in EnStem A neural differentiation medium (SCM017, Millipore) and treated with recombinant human IL-4 (50 ng/ml, r-huIL-4, Peprotech), r-huIL-4+ anti-IL-4 neutralizing antibody (αIL-4, 10 µg/ml, R&D Systems) or equal volumes of PBS for 24 h. Following fixation with 4% PFA, cultures were stained via immunocytochemistry.

### Immunocytochemistry (ICC)

CxV explants were fixed at 4–6 div for 10 min in 4% PFA (Sigma), washed with PBS and permeabilized for 10 min with 0.2% Triton-X in PBS. The following primary antibodies were used: Gad67 (Chemicon), GAP-43 (Abcam), GFAP (Sigma), mO4 (gift from J. Trotter), PKCγ (Santa Cruz Biotechnology), phospho-GAP-43 (Thermo Fischer), phospho-PKCγ (Biozol), PTEN Cascade Biosciences), PV (Swant), SMI-32 (Covance), and β-III-Tubulin (Tubb3, mouse, Covance; rabbit, Abcam). For human neuronal cultures IL-4R (BD Pharmingen), Homer-1/2/3 (Synaptic Systems), IRS1 (Abbexa), phospho-IRS1 (pIRS1, Abbexa), NeuN (Merck Millipore), PKCγ (Abcam), Neurofilament light chain (Abcam), MAP2 (Abcam), Synaptophysin (Synaptic Systems), and Tubb3 (BioLegend), were used in addition. After washing with PBS, incubation was performed for 1 h with Alexa-conjugated anti-mouse and anti-rabbit secondary antibodies (Life Technologies), Alexa 488-conjugated Phalloidin (Thermo Fisher) and dapi (Invitrogen).

### Reverse Transcription Quantitative Polymerase Chain Reaction (RT-qPCR)

Total RNA isolation was performed as previously described with minor modifications (Vogelaar et al. 2009). Briefly, after washing the cultures with PBS, explants and adhering axons were collected in lysis buffer, pooling 2–3 wells per sample. After RNA isolation using RNeasy Micro Kit (Qiagen) according to manufacturer’s instruction, samples were subjected to RNase-free DNaseI (Roche) and further purified using the RNeasy MinElute RNA cleanup kit (QIAGEN). cDNA synthesis was performed with the Superscript III First-Strand Synthesis System (Invitrogen) using a mix of random primers and Oligo(dT). Primers (Suppl. Table S1) were designed using the Beacon Designer Software (Premier Biosoft) and optimized on control brain cDNA to determine optimal primer concentration, annealing temperature and efficiency. qPCRs were run in 96-wells plates in the CFX96™ Real-Time PCR Detection System (Biorad). Levels of target genes were calculated in relation to housekeeping genes as previously described (Vogelaar et al. 2009).

### Microscopy

Light microscopic images from CxV explants were obtained using the Olympus IX51 and CellSense Dimension software (Olympus, XC30). Human differentiated neurons were imaged with the Keyence BZ-X710 fluorescent microscope for outgrowth analysis. Quantification of Tubb3^+^ neurites was performed in a double-blinded manner using the Simple Neurite Tracker plugin of Fiji (ImageJ) by tracking at least 50 neurites from at least 2 coverslips per condition. Statistical analysis (One-way ANOVA) was performed using the GraphPad Prism software. Data are representative of 2–3 independent experiments. For confocal microscopy, the Leica SP8 microscope with LAS X software (Leica) was used. Xyz-stacks with system optimized step size between the planes were acquired for each scanning sequence and 3–10 chosen optical planes were subsequently used for maximum projection.

## Results

### Long-Term CST Axon Cultures

In general, there is a lack of in vitro models for the regeneration of CNS projection axons. We therefore chose to develop CST axon cultures, based on previous peripheral DRG axon cultures (Vogelaar et al. 2009) and a short-term repulsion model (Liu et al. 2005). Cultivating explants from mouse motor cortex layer V (CxV), we were able to achieve long CST axon growth (Fig. 1, Suppl. Fig. S1, see “Materials and Methods”). We found that only CxV explants exhibited significant neurite outgrowth while explants of other layers displayed minor outgrowth (data not shown), corroborating the projection neuron phenotype of layer V. The axons survived for at least 2 weeks, reaching a length of more than 2.5 mm (Suppl. Fig. S1b).

Relevant differences were observed between CxV and DRG cultures. Close to the explant, cortical axons formed network-like patterns, whereas DRG axons grew mainly linearly (Fig. 1c). Further away from the explants, both DRG and CST axons segregated into distinguishable straight axons with clear growth cones. CxV axons were 1.8-fold thinner and displayed smaller growth cones than the DRG axons (Fig. 1d). Comparable observations were made in parasagittal spinal cord sections of YFP-H mice, which express yellow fluorescent protein (YFP) in the CST and dorsal columns (DC) (Carter et al. 2008; Feng et al. 2000), the latter representing ascending fibers arising from the DRGs. Interestingly, both axon types were smaller in absolute diameter in culture than their in vivo counterparts, however, the relative difference in diameter was comparable (Fig. 1d, f). In contrast to DRG axons, which grow several cm in culture (data not shown), CxV explant axons reached only 2–3 mm in length. Table 1 illustrates the lifespan of a representative CxV culture. The total distance the axons were able to grow varied per explant, depending on the time the explant needed to settle down after plating. To further assess the growth capacity of CxV cultures, we performed RT-qPCR for phosphatase and tensin homologue (PTEN) and suppressor of cytokine signaling 3 (SOCS3), which are known to restrict regenerative growth (Sun et al. 2011). Both molecules were highly expressed; moreover, PTEN immunoreactivity was readily detectible in the cultured axons, suggesting that this protein may be involved in the reduced capacity to grow (Suppl. Fig. S3).

**Table 1:** CxV lifespan

Div	Axons	Cells leaving explant	Viability
1	Start growing	Migrate	Risk of detachment
2	300–600 µm	Migrate	Explant shrinks ± 0.5 µm
3	Up to 1.5 mm	Stay in 300 µm area near explants	Steady axon growth
7	Up to 2.2 mm		Axon growth slows down
14	Up to 2.75 mm	Migrated cells die	Risk of axon degeneration

*div* days in vitro

**Fig. 2:**
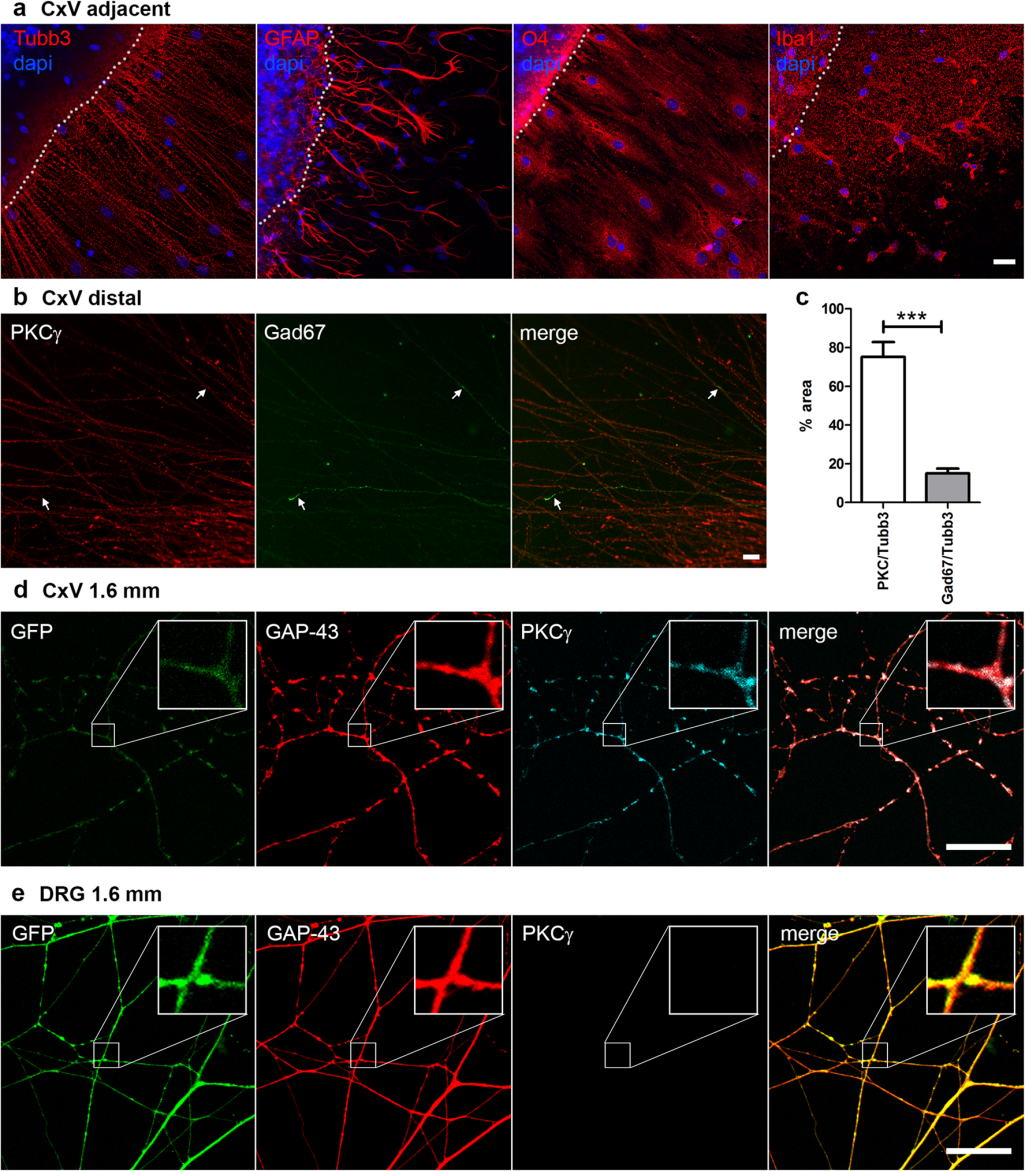
CxV explants mainly grow CST projection axons. **a** Immunocytochemistry of motor cortex layer V (CxV) explant cultures after 5 days in vitro for markers (red) of neurons (β-III-Tubulin, Tubb3), astrocytes (GFAP), oligodendrocytes (O4), and microglia (Iba1). Nuclei were stained with dapi (blue) and the edge of the explant delineated with white dots. **b** Distal CxV axons stained for Gad67 (green) and PKCγ (red) showing that the majority of the growing axons arise from CST neurons (green, arrows). **c** Quantification of the area of PKCy^+^ and Gad67^+^ axons relative to Tubb3 (*n* = 3 and images, resp.). **d**, **e** Immunocytochemistry of CxV (**d**) and DRG (**e**) explant cultures from a GFP (green) mouse at 1.6 mm distance from the explants. Axons were stained for GAP-43 (red), a marker for growing axons, and PKCγ (cyan), a marker for CST axons (boxed area enlarged). Scale bars: 25 µm. Statistics: unpaired *t*-test, *p* < 0.001

During the first 2 days in vitro (div) the explants shrunk, leaving some cell debris. This might be partially due to cell death, but was also caused by migration of cells (Figs. 1c, 2a). Migrated cells covered an area of up to 300 µm surrounding the explant in declining density (Suppl. Fig. S1a). The majority of cells leaving the explants were oligodendrocytes, whereas astrocytes stayed in close vicinity to the explants (Fig. 2a) and neurons only occasionally migrated. To characterize the types of axons extending from the CxV explants, we stained for parvalbumin (PV), a marker for a subgroup of GABAergic interneurons (Rudy et al. 2011)and non-phosphorylated neurofilaments (SMI-32), a marker for pyramidal neurons (Voelker et al. 2004; Wannier et al. 2005). We observed that the majority of axons were SMI-32^+^ CST axons, whereas only some PV^+^ axons were found (Suppl. Fig. S4). Using the more specific CST marker protein kinase C gamma (PKCγ) (Starkey et al. 2005) and the pan-GABAergic marker Gad67 (Rudy et al. 2011), we then quantified the relative contribution of CST and GABAergic axons. Whereas 75% of axons were PKCγ^+^, only 15% displayed Gad67 (Fig. 2b, c). These data suggest that the CxV cultures mainly represented CST projection axons, with a small contribution of GABAergic interneurons. Next, we compared the outgrowth of axons from DRG and CxV explants of mice ubiquitously expressing green fluorescent protein (GFP). Due to their small diameter, the GFP signal declined in the distal CST axons. The growth-associated protein GAP-43 was present in both types of axons, whereas PKCγ was only present in the CxV axons (Fig. 2d, e). Strikingly, GAP-43 and GFP were differently distributed in CST and PNS axons. Whereas the labeling in PNS axons was diffuse (Fig. 2e), the CST axons displayed discontinuous patterns (Fig. 2d). This was also observed for Tubb3 and SMI-32 (Fig. 2a, Suppl. Fig. S4). This was not due to degeneration, since brightfield microscopy showed that axons were morphologically intact and continued to grow for up to two weeks (Suppl. Fig. S1b). Therefore, we conclude that not only CST axon size and diameter, but also the structure of the CST cytoskeleton differed from DRG axons.

### IL-4R Signaling and CxV Growth Assay

**Fig. 3:**
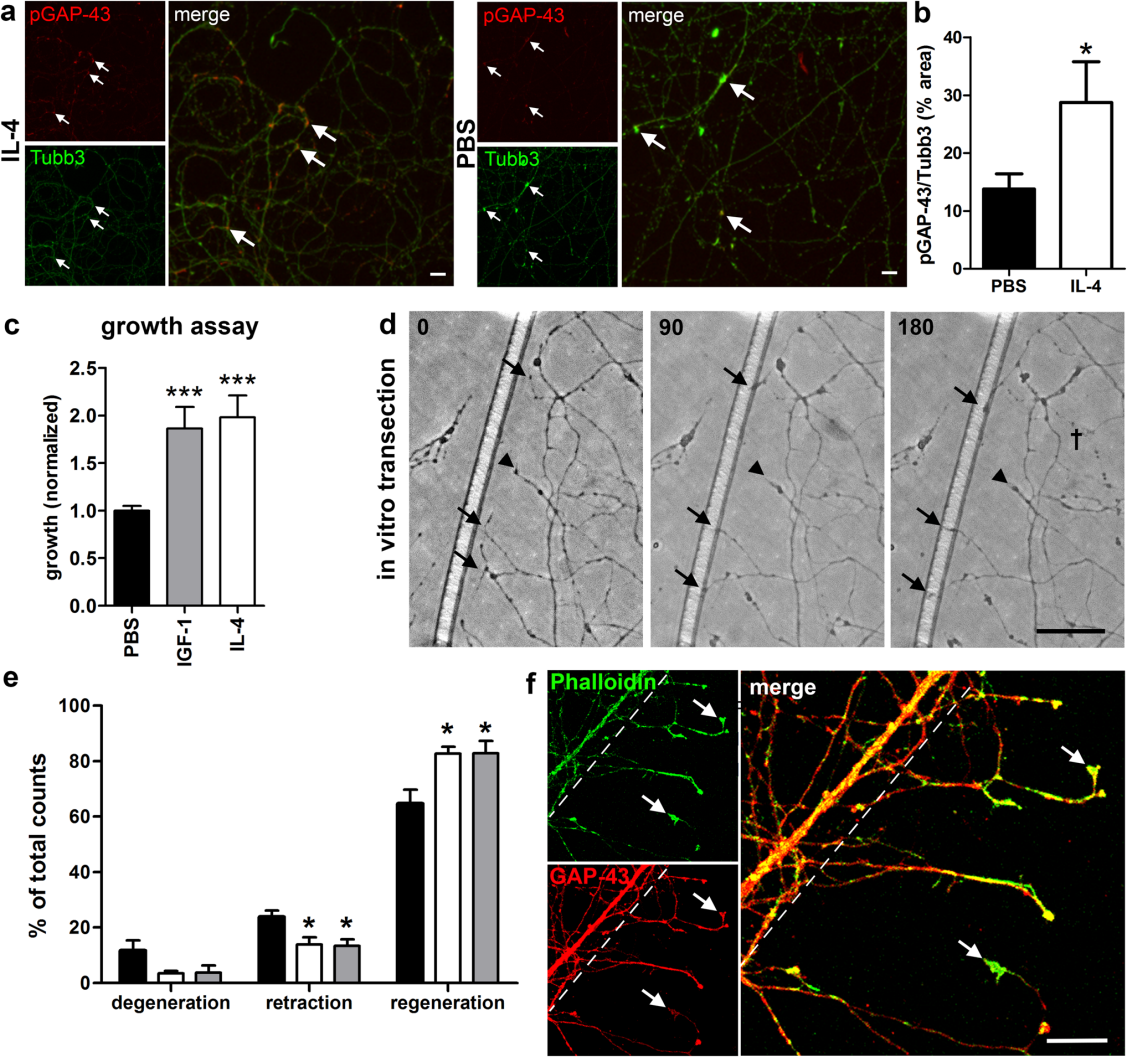
IL-4 increases regeneration of CST axons after transection. **a** Immunocytochemistry of CST axons for Tubb3 (green) and pGAP-43 (red) at 30 min after treatment with IL-4 (left panels) or PBS (right panels). **b** Quantification of IL-4-induced GAP-43 phosphorylation (area of pGAP-43^+^ pixels relative to Tubb3) (PBS *n* = 8; IL-4 *n* = 6). **c** CxV growth assay showing quantification induced axon outgrowth after treatment with IL-4 (*n* = 5) and IGF-1 (*n* = 4) compared to PBS controls (*n* = 12). **d** In vitro transection assay enables assessment of CST regeneration after treatment. Transection site visualized by scratch on plastic surface (left). Markings highlight regenerating (arrows), retracting (arrow heads) and degenerating (cross) axons. Axons were transected at approximately 1 mm distance to the explant (see Suppl. Fig. S2) and imaged at 0, 90, and 180 min post-transection. **e** Quantification of degeneration, retraction and regeneration after treatment with IGF-1 (gray bars, *n* = 5) and IL-4 (white bars, *n* = 4), compared to PBS controls (black bars, *n* = 5). **f** Immunocytochemistry of transected CST axons 120 min post-transection with Phalloidin (green) and GAP-43 (red) showing robust formation of new growth cones and thus regeneration in response to transection. Dashed line represents transection site. Scale bars: **a**, **f** 10 µm, **d** 20 µm. Statistics: **b** unpaired *t*-test; **c**, **e** one-way ANOVA with Tukey’s multiple comparison test *p < 0.05, ***p < 0.001

In order to confirm that the IL-4R signaling pathway is also active in CxV axons, we performed immunohistochemistry for phospho-GAP-43 (pGAP-43) and Tubb3 after 30 min of treatment with IL-4 (50 ng/ml) or PBS. Quantification of the pGAP-43^+^ signals in the distal axons compared to Tubb3 revealed a twofold upregulation of phosphorylation in IL-4-treated axons (Fig. 3a, b). We already showed that IL-4 is able to induce axon outgrowth (Vogelaar et al. 2018), however, we now compared its efficacy with known neurotrophic factors. Here we show that IL-4 enhanced neurite outgrowth from CxV explants to a similar extent as insulin-like growth factor-1 (IGF-1), a known neuronal growth factor (Koopmans et al. 2006) (Fig. 3c).

### A CST Regeneration Assay for Fast Screening of Growth-Promoting Treatments

Explant cultures present a great advantage over dissociated cell cultures because they grow long distances of more than 2 mm, enabling manual transection similar to the procedure described for DRGs (Verma et al. 2005; Vogelaar et al. 2009). In order to follow regeneration of individual CST axons, transections were performed about 1 mm distal to the explant (Suppl. Fig. S2). Regenerative events were quantified by scoring the formation of new growth cones and the extension of axons at 90 and 180 min post-transection (Fig. 3d). Fluorescent immunocytochemistry for filamentous actin (Phalloidin) and GAP-43 at 120 min post-transection confirmed the formation of new growth cones (Fig. 3f). Using IGF-1 to validate the model, we found a significant reduction of axon retraction accompanied by an increase of regeneration in comparison to PBS controls. The application of 50 ng/ml IL-4 caused a comparable significant increase of regeneration and decrease of axon retraction and degeneration (Fig. 3e). The effects on regeneration occurred mainly within the first 90 min post-transection, since data at 90 and 180 min were comparable (data not shown). Taken together, we here characterized a CNS explant culture system suitable as a regeneration model for CST axons, and showed that the effects of IL-4 on regenerative growth after injury are comparable to IGF-1.

### Interleukin-4 Stimulates Neurite Outgrowth of Human Stem Cell-Derived Neurons

**Fig. 4:**
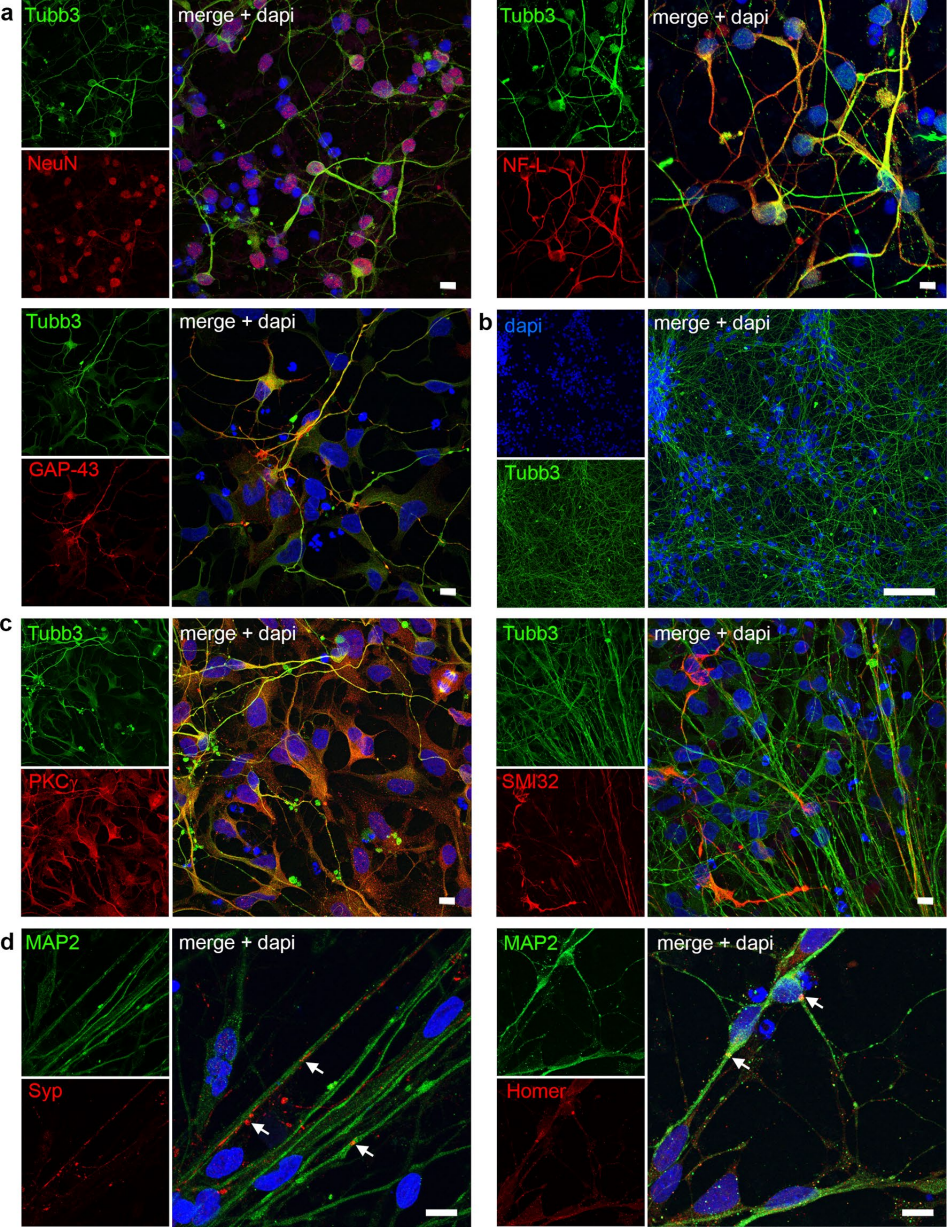
Human neurons in culture display CST markers. **a** Immunocytochemistry of human stem cell-derived neurons for neuronal lineage markers (NeuN, NF-L, GAP-43; all red) co-labeled with β-III-Tubulin (Tubb3, green). Nuclei labeled with dapi (blue) **b** Neuronal network formation after 3 weeks of differentiation. **c** Human neurons stained for CST marker PKCγ (red) and pyramidal neuron marker SMI-32 (red), co-labeled with Tubb3, respectively. **d** Synaptic contacts as shown by staining for synaptophysin (Syp, red) and Homer1 (red), with counterstaining for microtubule-associated protein 2 (MAP2, green). Scale bars: **a**, **c**, **d** 10 µm; **b** 100 µm

We next developed a human model for axon outgrowth that we could use to further explore the clinical translational potential of candidate SCI treatments. Human neural stem cells (hNSCs) were cultured in neural differentiation medium to generate neurons. Differentiated human neurons expressed a variety of neuronal markers including neurofilament light chain (NF-L), β-III-Tubulin (Tubb3), GAP-43, and NeuN, suggesting robust differentiation into mature neuronal cells (Fig. 4a). First signs of neurite outgrowth were already present after 5–7 days post-differentiation, and after 3 weeks, the cells formed a dense neuronal network (Fig. 4b). To further characterize neuronal subtypes present in culture, we stained for PKCγ, SMI-32 and PV. Interestingly, we found the majority of neurons expressing PKCγ at 3 weeks in vitro, indicating that the human neurons differentiated into CST-type neurons (Fig. 4c). A substantial subset of axons was also positive for SMI-32 (non-phosphorylated neurofilament-H, Fig. 4c), whereas PV^+^ axons were not observed (data not shown). At 3 weeks of differentiation, the neurons displayed synapse formation, as shown by immunocytochemistry for pre- and post-synaptic markers, Synaptophysin and Homer, respectively (Fig. 4d).

**Fig. 5:**
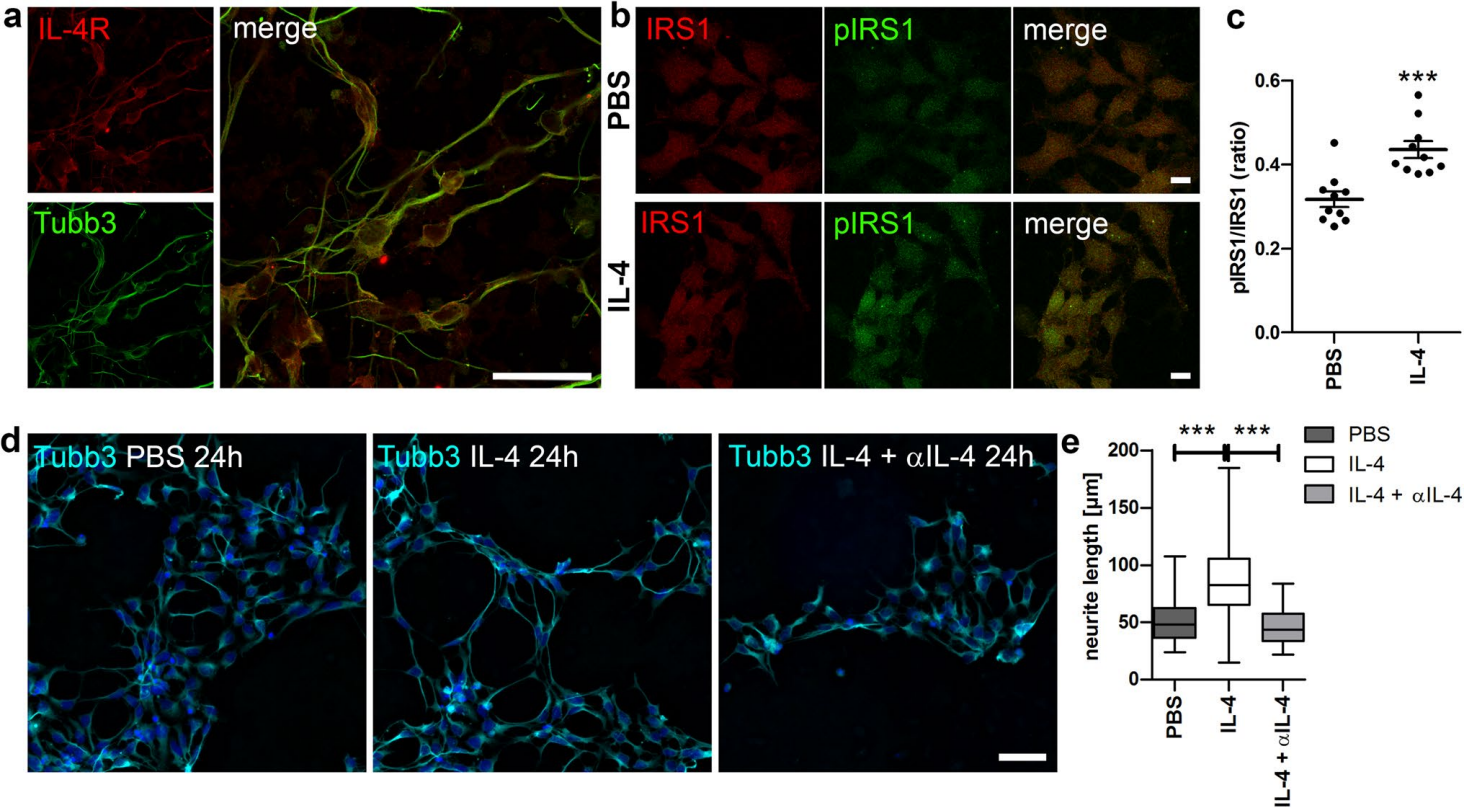
IL-4 induces neurite outgrowth of human neurons. **a** Human neurons express the IL-4Rα (red) as shown by overlap with the neuronal marker β-III-Tubulin (Tubb3) (green). **b** Exemplary images of IRS1 and pIRS stainings in PBS and IL-4 treated cultures. **c** Quantification of fluorescence intensity ratio of pIRS1 and IRS1 in Tubb3^+^ cells (*n* = 10 images per treatment). **d** Exemplary images of human neurons treated with IL-4, IL-4 + αIL-4 antibody, or PBS for 24 h stained with Tubb3 (cyan) and dapi (blue). **e** Quantification of treatment effects for 24 h shows a significant increase in Tubb3^+^ neurite outgrowth in IL-4-treated cultures that is abolished by αIL-4 (*n* = 100–150 neurons per treatment). Scale bars: **a**, **b** 10 µm, **d** 50 µm. Statistics: **c** unpaired *t*-test and **e** one-way ANOVA with Tukey’s multiple comparison test ***p < 0.001

Since we observed IL-4R expression on differentiated human neurons in this system (Fig. 5a), and IL-4 treatment increased the phosphorylation of IRS1 (Fig. 5b, c), previously identified as downstream of IL-4R (Vogelaar et al. 2018), we concluded that this human neuronal culture was a suitable model to analyze the IL-4-activity on human neurite outgrowth. After 6 days of differentiation, human neurons were treated with r-huIL-4 or PBS for 24 h. IL-4 induced a significant increase in neurite outgrowth, which was abolished by co-incubation with an αIL-4 neutralizing antibody (Fig. 5d, e). These data indicate that human neurons in culture respond directly to IL-4 by increasing their growth.

## Discussion

Among all tracts in the spinal cord, the CST is the tract with the lowest capacity to regenerate (Schiwy et al. 2009). Based upon our recently published observations of direct neuronal IL-4 signaling leading to repair of CST axonal swellings and functional recovery in the chronic phase of neuroinflammation (Vogelaar et al. 2018), and impaired regeneration in IL-4^−/−^ mice (Walsh et al. 2015), we hypothesized that IL-4 may directly stimulate CST regeneration. In fact, IL-4 has been reported to positively affect locomotor behavior after spinal cord injury (Fenn et al. 2014; Francos-Quijorna et al. 2016), but it is unclear whether the mechanisms are through indirect or direct effects on axons.

To investigate the effect of putative growth-promoting treatments on CST regeneration in more detail, we established murine and human CST culture models. So far, the closest in vitro approach to modeling CST axon regeneration has involved co-culturing of transgenic GFP-expressing sensorimotor cortex slices adjacent to longitudinal slices of WT thoracic spinal cord and analyzing the ingrowth of GFP^+^ axons (Pohland et al. 2015). However, these organotypic slice cultures are difficult to use for screening treatments because of methodological difficulties, such as the correct section plane of both explants, their survival, and the distance between them. Bagnard et al. have previously cultured E16 rat cortices and investigated growth cone behavior (Bagnard et al. 2001). Since cortical motor neurons are generated between E11-17 in mice and the migration to their final layer destination takes 24 h (Takahashi et al. 1999), complete cortical explants from E16 embryos are not specific for CST motor axons. Axonal growth in the spinal cord CST area occurs between P2 and P9 in rostro-caudal direction (Gianino et al. 1999). Therefore, by dissecting the cortical explants from neonatal animals up to P5, we ensured that the neurons in these explants were axotomized and regenerating, rather than continuing their embryonic development.

Here, we provide a straightforward mouse neonatal cortical explant culture, where we specifically cultivated layer V of the motor cortex only. Two CST markers, SMI-32 (Voelker et al. 2004; Wannier et al. 2005) and PKCγ (Starkey et al. 2005), were expressed by the majority of axons in the CxV culture. Furthermore, the growing axons were positive for the regeneration-associated protein GAP-43. We adapted the DRG and CxV culture conditions in terms of medium composition to achieve similar conditions allowing for comparison of PNS and CNS axons. Interestingly, CST axons were structurally different from PNS axons, displaying smaller growth cones, smaller diameters, and a less continuous cytoskeletal structure. However, the apparent interrupted appearance cannot be due to degeneration, since these axons grew for up to 2 weeks and were capable of regeneration after in vitro transection. A striking difference between DRGs and CxV explants was the total distance the axons were able to grow. DRG axons grew several cm and CxV only a few mm (qualitative observations), whereas in vivo, the peripheral nerves and spinal cord tracts are similar in length. Since CxV cultures displayed high expression of PTEN and SOCS3, which are known limiting factors in CNS regeneration (Sun et al. 2011), we conclude that the CxV cultures have a limited growth capacity, allowing stimulation by putative growth-promoting treatments.

Having characterized our model as primarily corticospinal, we then set out to validate it for the testing of regeneration-promoting treatments. The outgrowth assay showed that IL-4 caused a similar increase in axon growth as IGF-1, and phosphorylation of the growth-promoting protein GAP-43 was increased to a similar extent compared to our previously reported phosphorylation in dissociated cortical cultures (Vogelaar et al. 2018). Importantly, the novel axon transection assay provides an even more interesting screening method for putative regeneration-promoting treatments. This is the first model to show that CNS axons are capable of new growth cone formation after in vitro axotomy. An earlier study on retinal explants suggested failure of CNS regeneration (Verma et al. 2005), however, these were cultures of adult tissue, reducing the likelihood of growth per se. In our study, the axotomized CST axons displayed a robust increase in regeneration in response to IL-4, equaling IGF-1-induced regeneration. Previously, IL-4 effects on recovery were suggested to be mediated via myeloid cells (Francos-Quijorna et al. 2016). However, since non-neuronal cells stayed inside or in close vicinity to the explants in our model, we conclude that regeneration of the distal CST axons during the first few hours after axotomy is most likely due to direct effects of IL-4 on their intrinsic growth capacity.

Human and mouse IL-4 are strikingly different, with only 40% amino acid conservation. Accordingly, mouse IL-4 is unable to bind and stimulate the human IL-4R at physiological concentrations (Mueller et al. 2002). In order to test whether human IL-4 has comparable effects on human neurons, we differentiated hNSCs to neurons that were mainly corticospinal, as shown by the CST markers PKCγ and SMI32. Similar to the effects of mouse IL-4 on CST explants, human IL-4 was able to phosphorylate downstream signaling molecules and stimulate outgrowth of human neurons, suggesting that IL-4 has direct effects on neurite growth across species.

In conclusion, we developed a fast and reproducible culture model for the screening of treatments that promote regeneration of the CST, the spinal cord tract with the poorest regenerative capacity. Moreover, the ability to test growth-promoting treatments on human neurons is a significant step forward in the regeneration field. We demonstrate that IL-4 is able to directly stimulate CST neurite outgrowth and regeneration of mouse CxV explants and human CST neurons. This assay system represents a valuable new tool that would accelerate screening for treatments that increase the regenerative capacity of CST axons.

## Electronic supplementary material

Below is the link to the electronic supplementary material.

Supplementary file1 (DOCX 2392 kb)
