# Supplementary material for: Growth-Promoting Treatment Screening for Corticospinal Neurons in Mouse and Man
Source: Cell Mol Neurobiol. 2020 Mar 14;40(8):1327–38. doi: 10.1007/s10571-020-00820-7 (PMC7497511; doi:10.1007/s10571-020-00820-7)
Supplement: Supplementary file 1 — Supplementary file1 (DOCX 2392 kb) [file 10571_2020_820_MOESM1_ESM.docx]

**Growth-promoting treatment screening for corticospinal neurons in mouse and man**

Nicholas Hanuscheck^1*^, Andrea Schnatz^2^*, Carine Thalman^1*^, Steffen Lerch^1^, Yvonne Gärtner^1^, Micaela Domingues^1^, Lynn Bitar^1^, Robert Nitsch^3^, Frauke Zipp^1^, Christina F. Vogelaar^1,#^

^1^Department of Neurology, Focus Program Translational Neuroscience (FTN) and Immunotherapy (FZI), Rhine Main Neuroscience Network (rmn^2^), University Medical Center of the Johannes Gutenberg University Mainz, 55131 Mainz, Germany

^2^Institute for Developmental Biology and Neurobiology, Molecular Cell Biology, Johannes Gutenberg University Mainz, 55099 Mainz, Germany

^3^Institute for Translational Neuroscience, University Medical Center, Westfälische Wilhelms-University Münster, Albert-Schweitzer-Campus, 48149 Münster, Germany.

*Equally contributing first authors

**^#^Corresponding author:**

Dr. Christina Francisca Vogelaar

University Medical Center of the Johannes Gutenberg University Mainz,

55131 Mainz, Germany

Email: [tineke.vogelaar@unimedizin-mainz.de](mailto:tineke.vogelaar@unimedizin-mainz.de)

**Supplementary Material**

**Supplementary table 1: RT-qPCR primer properties**

Abbreviations: GAP-43, growth-associated protein-43; PTEN, phosphatase and tensin homologue; RPS29, ribosomal protein small subunit 29; SOCS3, suppressor of cytokine signaling 3

| Gene | Primer (fw) | Primer (rev) | Conc. (nM) | Temp. (°C) | Efficiency (%) |
| --- | --- | --- | --- | --- | --- |
| GAP-43 | AGTGCCCGACAGGATGAG | CAGGACAGGAGAGGAAACTTC | 200 | 55 | 94.5 |
| PTEN | GTAATGACTGCTCCATCTC | AGGTAAGGTGTTGACTGA | 200 | 58 | 109.5 |
| RPS29 | CAAATACGGGCTGAACAT | GTCGCTTAGTCCAACTTAA | 200 | 58 | 101.5 |
| SOCS3 | GCTTTGATTTGGTTTGAT | GGGAGTGGTTATTTCTTT | 400 | 60 | 93.8 |

**

**

**Figure S1: CxV explant cultures**

**(a)** Merged image of a representative CxV culture at 3 days *in vitro*, showing migration of cells in the vicinity of the explant (up to 300 µm), with a dense network of axons that segregate to single straight axons distal from the explant. **(b)** CxV explants and axons survived up to 14 days *in vitro*, with axons extending for >2 mm distally. Scale bar: 100 µm. Statistics: one-way ANOVA with Tukey’s multiple comparison test, *** p < 0.001.

**
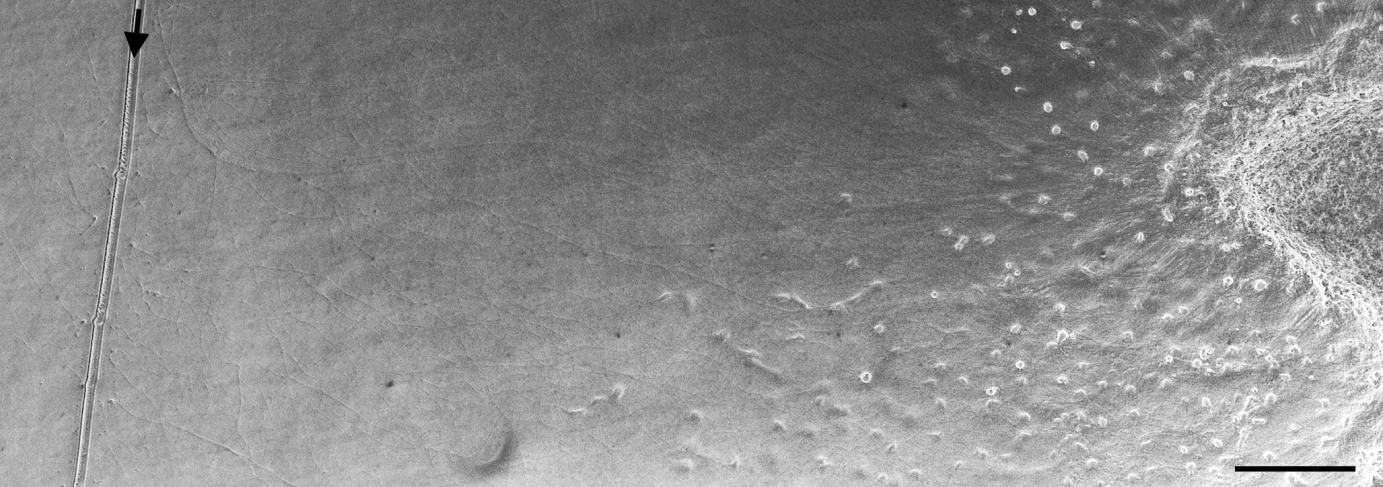
**

**Figure S2: *In vitro* transection paradigm**

Low resolution image of transection site (arrow) illustrating distance from the explant. Scale bar: 200 µm.


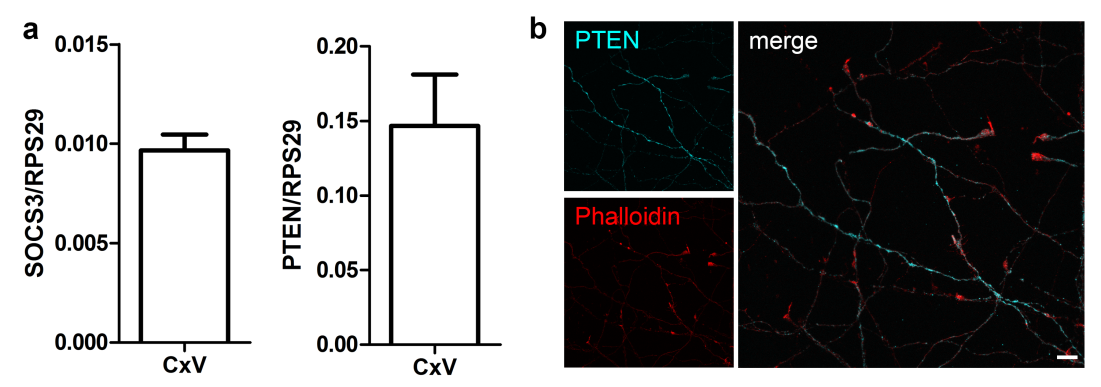


**Figure S3: Expression of growth-limiting molecules in CxV cultures**

**(a)** RT-qPCR analysis of SOCS3 and PTEN compared to the housekeeping gene RPS29. **(b)** Immunocytochemistry on CxV axons with PTEN (cyan) and the axonal F-actin marker Phalloidin (red). Scale bar: 5 µm


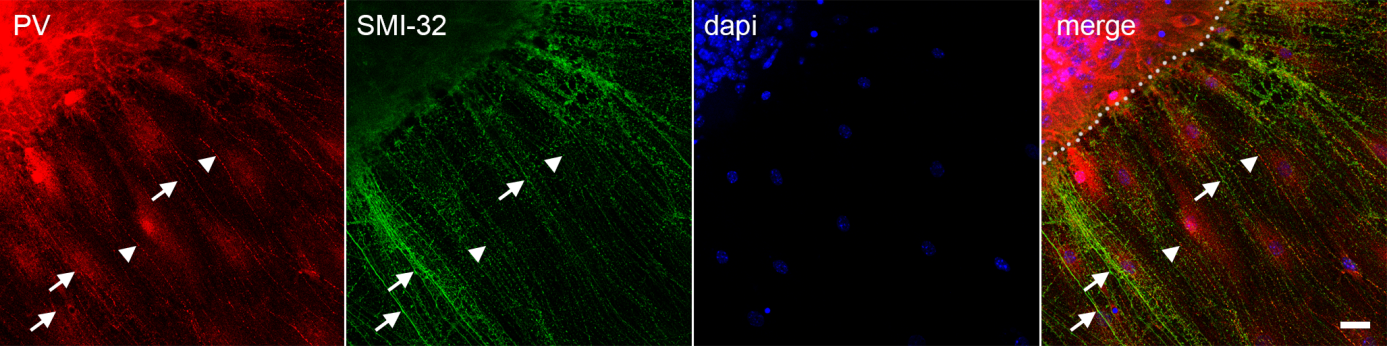


**Figure S4: CxV explants mainly grow projection axons**

Immunocytochemistry of motor cortex layer V (CxV) explant cultures after 5 days *in vitro* stained for parvalbumin (PV, red, arrowheads) and non-phosphorylated neurofilament (SMI-32, green, arrows), a marker for pyramidal neurons, showing that the majority of the growing axons arise from CST neurons. Nuclei were stained with dapi (blue) and the edge of the explant delineated with white dots. Scale bar: 25 µm.
